# Incidence of splenic malignancy and hemangiosarcoma in dogs undergoing splenectomy surgery at a surgical specialty clinic: 182 cases (2017–2021)

**DOI:** 10.1371/journal.pone.0314737

**Published:** 2024-12-03

**Authors:** Brigita Ziogaite, Elena T. Contreras, Jason E. Horgan

**Affiliations:** 1 Leader Animal Specialty Hospital, Cooper City, Florida, United States of America; 2 Shreiber School of Veterinary Medicine of Rowan University, Mullica Hill, New Jersey, United States of America; Ross University School of Veterinary Medicine, SAINT KITTS AND NEVIS

## Abstract

The objectives of this study were to evaluate the risk and predictive factors of splenic malignancy and hemangiosarcoma in dogs undergoing splenectomy at a surgical specialty clinic. Medical records, hematologic results, surgical reports, and histopathologic results from 182 dogs that underwent splenectomy for the treatment of splenic masses or nodules were reviewed retrospectively. The majority of dogs (57.7%) had benign splenic diagnoses with no malignancy. Hemangiosarcoma was diagnosed in 32.4% of the dogs. A final multivariable model indicated that thrombocytopenia, anemia, and a smaller diameter of the largest splenic nodule were risk factors for hemangiosarcoma (P<0.001), and hemoperitoneum (P = 0.01) was an additional risk factor when nodule diameter was not evaluated. There were 91 dogs that had hemoperitoneum, and 60.4% of those dogs had malignant splenic lesions. Of the 33 dogs that underwent a splenectomy for incidentally identified splenic lesions, 93.9% had benign splenic lesions. Breed size was not a significant predictor of splenic malignancy risk; however, all 6 of the German shepherds included in the study had a hemangiosarcoma diagnosis. Overall prevalence of splenic malignancy including HSA may be overestimated in some canine populations.

## Introduction

Canine splenic masses are commonly encountered in veterinary practice. Size and distribution of the masses range from small focal or multifocal nodules to large cavitated masses, and they originate from a variety of tissues including lymphoid, vascular, fibrous, smooth muscle, and endothelial tissues. Benign splenic masses are usually diagnosed as hemangiomas, hematomas, or lymphoid hyperplasia [1–3], while the most commonly reported malignant tumor of the spleen is hemangiosarcoma (HSA) [2–6]. Based on appearance and clinical presentation, HSA and other malignant splenic lesions can be indistinguishable from benign splenic lesions; however, it is important to predict the likelihood of HSA or malignancy due to the poor prognosis associated with these diagnoses.

To better understand the likelihood of splenic malignancy, studies have explored numerous factors associated with benign and malignant splenic masses [7–9]. Factors such as breed, gender, neuter status, age, body size, hematologic values, mass-to-splenic volume ratio, spleen-to-body-weight percentage, and number of lesions present on the spleen have been investigated with variable results [7,10–12]. Presence of hemoperitoneum, anemia, thrombocytopenia, macroangiopathic hemolysis with presence of schistocytes, disseminated intravascular coagulation, and low total solids concentration have all been reported as associated with HSA [12–14].

One of the most common considerations when making clinical decisions regarding the likelihood of splenic malignancy and HSA when a splenic lesion is found, is the “double two-thirds” rule that states that approximately two thirds of splenic masses in dogs with splenomegaly are malignant, and two thirds of those are diagnosed as HSA [13]. This rule is often applied to all splenic masses with and without hemoperitoneum and is strongly established in clinical settings. Some recent studies [7,8,15] have not aligned with these early findings; nevertheless, splenectomy is typically performed as a treatment for patients with a splenic mass, although in most cases, it is performed without knowing if the mass is benign or malignant.

Guidelines regarding prognostic indicators for splenic malignancy and HSA warrant further investigation in different populations in different time periods. Preoperative diagnostics are continually advancing, canine demographics and breed distributions might be changing, and the histopathological findings of splenic masses and clinical patterns may differ from those established decades ago. Furthermore, patient and client populations might differ in non-university settings, and such critical decisions and guidelines must be based on risk suited to the setting. For instance, the economic burden of splenectomy surgery combined with a poor prognosis for an HSA diagnosis, can result in the unnecessary euthanasia of patients with benign splenic masses, especially in emergency settings where owners must make rapid decisions [16]. Splenectomy for benign masses can also result in shortened lifespans, as perioperative complications can arise and result in death [17–19]. Thorough knowledge of splenic pathology, risk factors for malignancies, and continuous reevaluation of data are needed to aid in the decision-making process for both clinicians and owners.

The objective of this study was to update the literature regarding the likelihood of malignant splenic neoplasia and HSA in dogs undergoing splenectomy after splenic nodules or masses were identified at a surgical specialty clinic. Our secondary objective was to identify factors predictive of HSA in this population of dogs. We hypothesized that, of the dogs undergoing splenectomy due to splenic lesions diagnosed at a specialty surgical practice, less than 66.7% of the splenic lesions would be histologically identified as neoplastic, and less than 44% would be histologically identified as HSA.

## Methods

### Case identification

An electronic medical records search was performed to identify dogs that underwent splenectomy surgery at Leader Animal Specialty Hospital between September 2017 to July 2021. Dogs with or without hemoperitoneum were included in the study if they had singular or multiple splenic masses or nodules and had a splenectomy performed as treatment for the splenic lesions. All surgeries were performed by board certified veterinary surgeons (ACVS diplomates), a residency trained surgeon, or surgical residents. A splenectomy surgical report and a complete histopathology report for the splenic nodule(s) or mass(es) tissues excised during splenectomy were required for inclusion. Dogs were excluded if they had a history of traumatic splenic rupture, immune mediated thrombocytopenia (IMTP), splenic torsion, splenic abscessation, or gastric dilatation-volvulus (GDV) leading to splenic vascular disruption.

### Data collection

Data collected from the medical records included dog’s age at splenectomy, breed, sex, neuter status, body weight, hematocrit (HCT), platelet count (PLT), other diagnostics, presence of nontraumatic hemoperitoneum, presence of pulmonary metastasis, perioperative anesthetics, documented reason for splenectomy, number and diameter of splenic nodules or masses, splenic histologic diagnosis, and successful survival to discharge.

Breeds were self-reported by the owner and recorded in the medical record. Breeds that were represented by more than five dogs were analyzed as individual breeds within a breed-size category. All other breeds, including dogs reported as mixed-breed dogs, were grouped by size into five additional categories: toy (1.0–4.9 kg), small (5.0–9.9 kg), medium (10.0–17.9 kg), large (18.0–35.9 kg), and extra-large (36.0 kg and higher). Therefore, a breed-size category was created that included breeds represented by more than 5 dogs and the 5 size categories.

HCT and PLT were obtained from the complete blood count (CBC) preoperative laboratory values. If a preoperative CBC was not available in the records, packed cell volume (PCV) on the day of surgery was substituted for the HCT. Both HCT and PLT were classified into categories based on the reference intervals at the surgical specialty clinic. HCT was classified into three categories: low (< 33%), normal (33%–56%), and high (> 56%). Platelet count was classified into 5 categories: very low (0 – 59K/uL), low (60–117), low normal (118–249), normal (250–490), and high (>490).

Histopathologic splenic diagnoses were retrieved from veterinary pathologists’ (ACVP diplomates) histopathology reports from the veterinary diagnostic laboratories where the splenic tissues were submitted post-operatively. Diagnoses were categorized as hemangiosarcoma (HSA), splenic malignancy including HSA, splenic malignancy other than HSA, and benign with no splenic malignancy. The presence of nontraumatic hemoperitoneum was confirmed with medical record documentation of peritoneal blood at or near the time of splenectomy. The number and diameter of splenic nodules or masses were collected from surgery reports. If multiple measurements from multiple lesions were identified, the diameter of the largest nodule or mass was used. Splenectomies performed due to incidental findings of splenic abnormalities intraoperatively during an unrelated surgery, were recorded and categorized as non-primary surgeries, while splenectomies performed during a scheduled splenectomy surgery were categorized as primary surgeries. Pulmonary metastatic disease diagnosis was based on the presence or absence of pulmonary nodule(s) on preoperative thoracic radiographic or computerized tomography (CT) imaging and recorded based on the veterinary radiologists’ (ACVR diplomates) reports when available. A dog was considered to be discharged successfully if the dog was released with standard post-operative recovery instructions. A dog was considered to be discharged unsuccessfully if the dog was euthanized or died after surgery but prior to discharge, or if the dog was released to the owner at the owner’s request to be euthanized at home or at another clinic.

### Statistical analysis

Data were described and analyzed using STATA version 14.2 (StataCorp. 2015. College Station, TX, USA). Descriptive statistics were calculated, and categorical data were expressed as frequencies and percentages, whereas continuous data were expressed as medians and ranges. The Shapiro-Wilk test was used to assess normalcy of continuous data (PLT and HCT); because of non-normalcy, the Wilcoxon rank sum test was used to compare median PLT and HCT in dogs diagnosed with HSA compared to median PLT and HCT in dogs with a benign diagnosis. Logistic regression modeling was used to assess the association between variables (age, sex, breed-size group, primary surgery, PLT, HCT, presence of hemoperitoneum, thoracic metastasis, number of splenic nodules, diameter (cm) of largest splenic nodule or mass) and diagnosis of malignancy and diagnosis of HSA. Variables with a liberal significance threshold of P<0.20 in univariable analyses were considered for inclusion in the final multivariable model. The Hosmer-Lemeshow statistic was used to test the overall fit of the model. The area under the ROC curve was used to evaluate the discriminatory ability of the model to predict HSA. Statistical significance was set at P< 0.05.

### Ethics statement

This manuscript was retrospective in nature and utilized data from clinical patients. All data was anonymized prior to use. Given the retrospective nature of this manuscript, prospective approval from the Institutional Animal Care and Use Committee (IACUC) was not required, as it was deemed not necessary by a university IACUC.

## Results

Initial record screening resulted in the identification of 206 dogs that underwent splenectomy during the study period. Of these dogs, 182 dogs met the criteria for study inclusion. The median age of dogs was 11 years (range 3 to 17 years). There were 84 females, 4 of which were intact, and there were 98 males, 9 of which were intact. The median body weight was 22.2 kg (range 1.2 to 53.2 kg). There were 54 breeds reported by owners. Mixed-breed dogs were most commonly reported (41/182, 22.5%), and these 41 dogs were reassigned to the size categories within the breed-size category. There were 5 breeds that consisted of more than 5 dogs (Table 1).

**Table 1:** Breed-size category and hemangiosarcoma diagnosis.

Breed-size category	Number (%) of dogs	Number (% of breed-size category) of dogs with HSA
Labrador retriever	15 (8.2%)	8 (53.3%)
Boxer	12 (6.6%)	5 (41.7%)
Bull terrier	10 (5.5%)	2 (20.0%)
German shepherd	6 (3.3%)	6 (100%)
Golden retriever	6 (3.3%)	4 (66.7%)
Extra-large (≥ 36.0 kg)	12 (6.6%)	3 (25.0%)
Large (18.0–35.9 kg)	36 (19.8%)	6 (16.7%)
Medium (10.0–17.9 kg)	37 (20.3%)	13 (35.1%)
Small (5.0–9.9 kg)	32 (17.6%)	8 (25.0%)
Toy (1.0–4.9 kg)	16 (8.8%)	4 (25.0%)
TOTAL	180	59 (32.8%)

Benign diagnoses with no malignancy were made in a total of 105 dogs (57.7%), while diagnoses of splenic malignancy including HSA were made in a total of 77 dogs (42.3%). HSA was diagnosed in 59 dogs (32.4%). A splenic malignancy other than HSA occurred in 21 dogs (11.5%); 2 of these dogs had a concurrent diagnosis of both HSA and lymphoma, and 1 of these dogs had a diagnosis of HSA or histiocytic sarcoma. Splenic malignancies other than HSA included liposarcoma (n = 2), lymphoma (n = 6), carcinoma (n = 1), splenic sarcoma (n = 8), and plasma cell tumor (n = 1) (Table 2).

**Table 2:** Histologic diagnoses of 182 dogs that underwent splenectomy.

Diagnosis	Number (%) of dogs	Number (%) of dogs with hemoperitoneum	Number (%) of dogs with non-primary splenectomy	Number (%) of dogs not surviving to discharge
Hemangiosarcoma^1^	59 (32.4%)	44 (24.2%)	2 (1.1%)	7 (3.8%)
Other malignancy not HSA	18 (9.9%)	11 (6.0%)	0 (0.0%)	1 (0.5%)
*Other malignancy and HSA* ^1^	*3 (1*.*6%)*	*2 (1*.*1%)*	*0 (0*.*0%)*	*0 (0*.*0%)*
Benign and no malignancy	105 (57.7%)	36 (19.8%)	31 (17.0%)	8 (4.4%)
TOTAL	182 (100%)	91 (50%)	33 (18.1%)	16 (8.8%)

^1^The one dog with a diagnosis of HSA or histiocytic sarcoma is included in this category.

Hemoperitoneum was present in 91 (50%) dogs. Of these 91 dogs, 55 (60.4%) had a splenic malignancy including HSA, while 36 of the dogs (39.6%) with hemoperitoneum had no malignancy. Of the 91 dogs with hemoperitoneum, 44 had HSA (Table 2).

The number of splenic masses or nodules was recorded for 180 (98.9%) of the dogs. The 180 dogs had one (n = 131), two (n = 13), three (n = 2) or multiple (n = 34) nodules. The 2 dogs with three nodules were reclassified as having multiple nodules. Diameter of the largest nodule was recorded for 143 dogs (78.6%); median diameter was 9.2 cm (range 0.5 cm to 40 cm).

Thirty-three of the dogs (18%) had a splenectomy performed as a non-primary surgery due to an incidental finding of a splenic nodule or mass during another surgery. Of these 33 dogs, 31 (93.9%) did not have a splenic malignancy. Only 2 (6.0%) of the 33 dogs had an HSA. None of the dogs that had a splenectomy due to incidental findings during surgery, had a non-HSA splenic malignancy (Table 2).

There was no evidence of thoracic metastasis in 172 (95%) of the dogs. Evidence of thoracic metastasis was only found in 3 dogs (2%), and they had splenic diagnoses of HSA, lymphoma, and hematoma. For the dog with a diagnosis of splenic hematoma, the CT report stated that pulmonary nodules were suspected to represent metastatic neoplasia, though osseous metaplasia was another differential. Thoracic radiograph interpretation was inconclusive for 6 dogs, and neither radiographs nor a report was located in the medical record for 1 dog. A veterinary radiologist (ACVR diplomate) report was located in the medical records for 176 (97.2%) of the dogs; for 5 of the dogs, image interpretation was performed by one of the surgeons.

There were 166 (91%) dogs that survived to discharge. Of the 16 dogs (8.8%) that were not successfully discharged, 7 had an HSA diagnosis, 1 had a carcinoma, and 8 had no malignancy diagnosed (Table 2). There were 10 additional dogs that were euthanized or died intraoperatively or immediately postoperatively; however, these 10 dogs were not included in the 182 dogs of this study cohort and analyses, as the owners did not consent to splenic histopathology.

**Fig 1:**
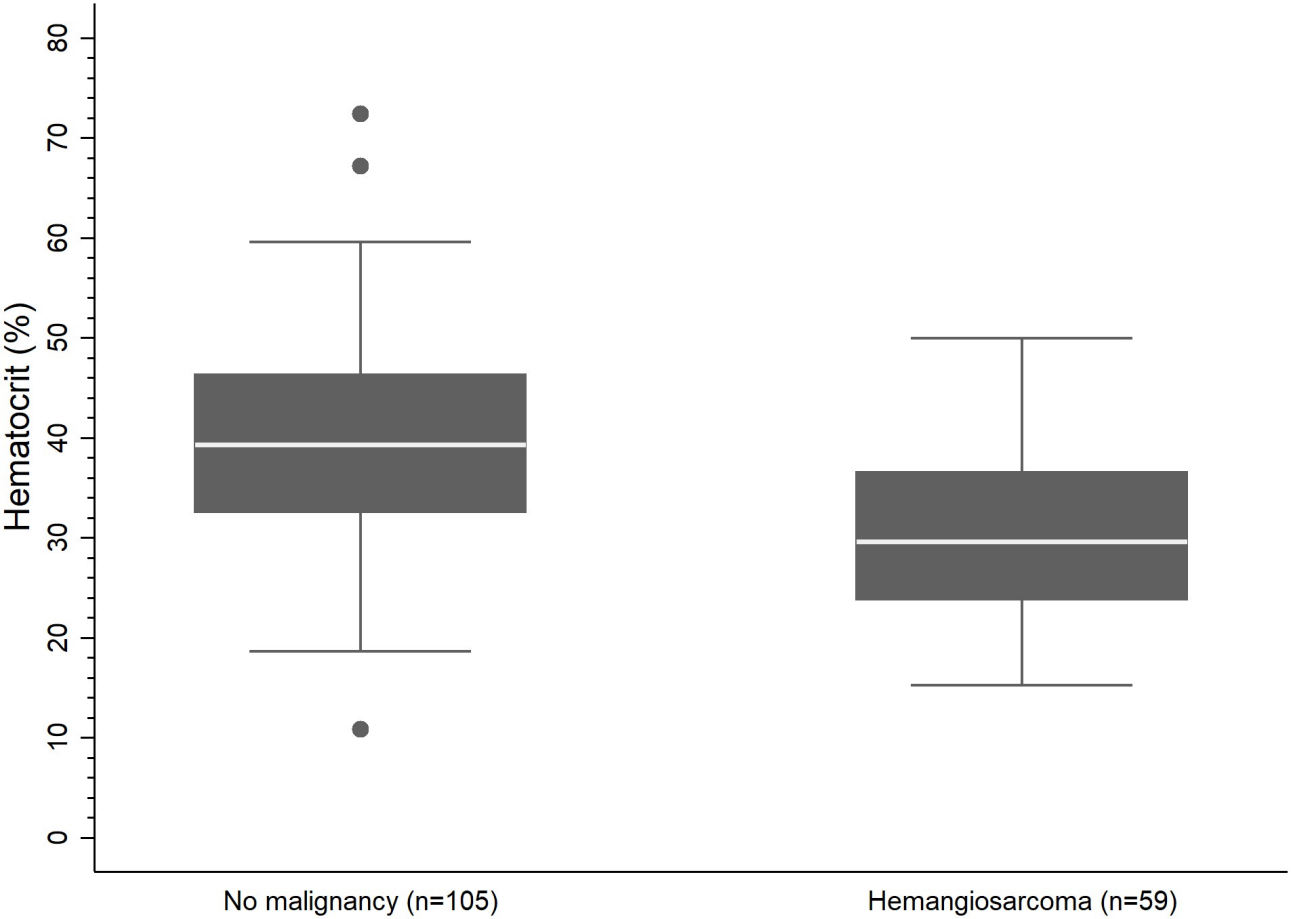
Boxplot comparison of hematocrit (HCT) percentages in dogs with and without hemangiosarcoma (HSA). Boxplot illustrates the distribution of HCT percentages among dogs diagnosed with hemangiosarcoma (n = 59) as compared to dogs with a benign splenic diagnosis and no malignancy (n = 105). The median and interquartile ranges are represented by horizontal lines and shaded boxes, respectively. Whiskers extend to the 5^th^ and 95^th^ percentiles. Dogs with HSA showed significantly lower HCT percentages as compared to dogs with a benign splenic diagnosis and no malignancy. (Wilcoxon rank sum test, P<0.001).

The median HCT was 36% (range 10.8%–72.4%). There were 2 dogs for which full bloodwork was not reported in the records. For those 2 dogs, PCV on the day of surgery was used in analyses. A low HCT was reported in 66 dogs (36.3%), while 110 dogs (60.4%) had a normal HCT, and 6 dogs (3.3%) had a high HCT. No dogs with a high HCT had a splenic malignancy. HCT values of dogs with a diagnosis of HSA (n = 59, median 29.6%, range 15.2% to 49.9%) were significantly lower (P<0.001) when compared to HCT values of dogs with a benign diagnosis and no malignancy (n = 105, median 39.3%, range 10.8% to 72.4% (Fig 1).

**Fig 2:**
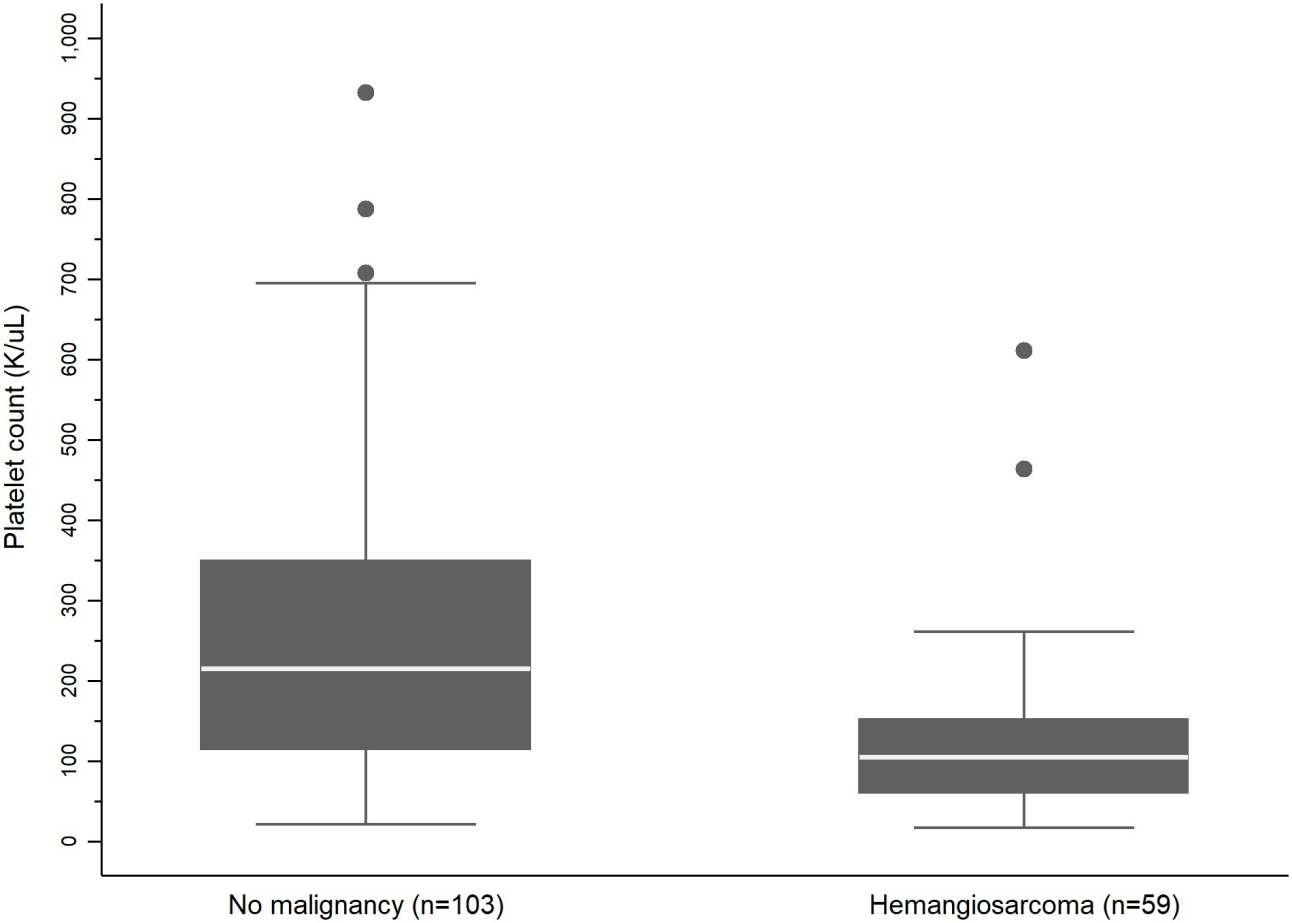
Boxplot comparison of platelet count (PLT) in dogs with and without hemangiosarcoma (HSA). Boxplot illustrates the distribution of PLT (K/UL) among dogs diagnosed with hemangiosarcoma (n = 59) as compared to dogs with a benign splenic diagnosis and no malignancy (n = 105). The median and interquartile ranges are represented by horizontal lines and shaded boxes, respectively. Whiskers extend to the 5^th^ and 95^th^ percentiles. Dogs with HSA showed significantly lower PLT percentages as compared to dogs with a benign splenic diagnosis and no malignancy. (Wilcoxon rank sum test, P<0.001).

The median PLT was 156K/uL (range 17 – 932K/uL) (Table 3). Of the 55 dogs with normal or high PLT, only 4 had HSA. PLT counts of dogs with a diagnosis of HSA (n = 59, median 105, range 17 to 59K/uL) were significantly lower (P<0.001) when compared to PLT counts of dogs with a benign diagnosis and no malignancy (n = 103, median 215, range 21 to 932K/uL) (Fig 2).

**Table 3:** Platelet counts and splenic malignancy.

Platelet count category	Number (%) of dogs	Number (%) of dogs with HSA	Number (%) of dogs with *NO* splenic malignancy
Very low (0-59K/uL)	30 (16.7%)	14 (7.8%)	13 (7.2%)
Low (60-117K/uL)	38 (21.1%)	22 (12.2%)	13 (7.2%)
Low normal (118-249K/uL)	57 (31.7%)	19 (10.6%)	34 (18.9%)
Normal (250-490K/uL)	44 (24.4%)	3 (1.7%)	34 (18.9%)
High (>490K/uL)	11 (6.1%)	1 (0.5%)	9 (5.0%)
TOTAL	180	59 (32.8%)	103 (57.2%)

Univariable logistic regression modeling identified six variables with liberally significant (P<0.20) association with HSA diagnosis: breed-size (P = 0.12, Table 1), PLT, HCT, primary surgery, presence of hemoperitoneum, and diameter (cm) of largest splenic nodule or mass. These variables were further evaluated using multivariable regression modeling. The final multivariable model for risk of HSA retained three significant risk factors, PLT, HCT, and diameter of largest splenic nodule or mass (Table 4). The final model fit the data well based on the Hosmer-Lemeshow statistics (χ^2^ = 8.23, P = 0.41) and showed good discrimination (area under the ROC curve = 0.81). Controlling for other variables in the model, the odds of HSA diagnosis were, on average, 21.4 times higher (95% CI 3.97 to 115.27, P<0.001) in dogs with low PLT (60K-117K u/L) as compared to dogs with normal PLT. The odds of HSA diagnosis were, on average, 3 times higher (95% CI to 1.27 to 7.51, P = 0.01) in dogs with a low HCT (<33%) as compared to dogs with a normal HCT. No dogs with a high HCT had HSA. For each 1 cm increase in the diameter of the largest splenic nodule, the odds of HSA diagnosis decreased, on average, by 10% (OR = 0.90, 95% CI 0.84 to 0.97, P = 0.01).

**Table 4:** Summary of multivariable analysis and hemangiosarcoma diagnosis.

Categorical variable	Level	Number	P-value	Coefficient	Odds ratio (OR)	OR 95% Confidence Interval
**Platelet group** *	Very low (0-59K u/L)	30	0.1	4.49	4.43	0.75 to 26.08
	Low (60K-117K u/L)	38	<0.001	3.06	21.4	3.97 to 115.27
	Low normal (118K–249K u/L)	57	0.01	2.12	8.35	1.65 to 42.38
	Normal (250K-490K u/L)	44	reference	reference	reference	reference
	High (>490K u/L)	11	0.21	1.76	5.83	0.38 to 89.88
**Hematocrit** *	Low (<33%)	66	0.01	1.13	3.09	1.27 to 7.51
	Normal (33%–56%)	110	reference	reference	reference	reference
	High (>56%)	6	*perfect predictor of no hemangiosarcoma*			
**Diameter of largest nodule** *	centimeters	143	0.01	-0.1	0.9	0.84 to 0.97
**Hemoperitoneum**	No	91	reference	reference	reference	reference
	Yes	91	0.06	0.92	2.52	0.97 to 6.55

* significant P-value at P< 0.05.

Because some dogs (n = 39) did not have splenic nodule diameters recorded in their records, logistic regression modeling was also performed excluding diameter as a variable. The final multivariable model for risk of HSA retained three significant risk factors, PLT, HCT, and hemoperitoneum (Table 5). The final model fit the data well based on the Hosmer-Lemeshow statistics (χ^2^ = 6.81, P = 0.45) and showed good discrimination based on performance under the ROC curve (AUC = 0.79). Controlling for other variables in the model, the odds of HSA diagnosis were, on average, 2.9 times higher (95% CI 1.28 to 6.37, P = 0.01) in dogs with hemoperitoneum as compared to dogs without hemoperitoneum.

**Table 5:** Summary of multivariable analysis and hemangiosarcoma diagnosis (without diameter included).

Categorical variable	Level	Number	P-value	Coefficient	Odds ratio	OR 95% Confidence Interval
**Platelet group** *	Very low (0-59K u/L)	30	0.02	1.69	5.43	1.28 to 23.05
	Low (60K-117K u/L)	38	<0.001	2.5	12.13	3.03 to 48.54
	Low normal (118K–249K u/L)	57	0.01	1.84	6.27	1.63 to 24.11
	Normal (250K-490K u/L)	44	reference	reference	reference	reference
	High (>490K u/L)	11	0.48	0.88	2.42	0.21 to 28.12
**Hematocrit** *	Low (<33%)	66	0.03	0.84	2.23	1.11 to 4.86
	Normal (33%–56%)	110	reference	reference	reference	reference
	High (>56%)	6	*perfect predictor of no hemangiosarcoma*			
**Hemoperitoneum** *	No	91	reference	reference	reference	reference
	Yes	91	0.01	1.05	2.85	1.28 to 6.37

* significant P-value at P< 0.05.

The following variables were not associated with HSA diagnosis: sex (P = 0.38), number of splenic nodules (P = 0.86), thoracic metastasis (P = 0.99), and age (P = 0.64).

When evaluating all splenic malignancy diagnoses including HSA in a multivariable model, logistic regression modeling identified five variables for inclusion in the model: PLT, HCT, primary surgery, presence of hemoperitoneum, and diameter (cm) of largest splenic nodule or mass (Table 6). The final model fit the data well based on the Hosmer-Lemeshow statistics (χ^2^ = 8.55, P = 0.38) and showed good discrimination (area under the ROC curve = 0.81).

**Table 6:** Summary of multivariable analysis and splenic malignancy including hemangiosarcoma.

Categorical variable	Level	Number	P-value	Coefficient	Odds ratio (OR)	OR 95% Confidence Interval
**Hematocrit** *	Low (<33%)	66	0.004	1.28	3.59	1.51 to 8.54
	Normal (33%–56%)	110	reference	reference	reference	reference
	High (>56%)	6	*perfect predictor of no malignancy*			
**Diameter of largest nodule** *	centimeters	143	0.002	-0.11	0.9	0.84 to 0.96
**Hemoperitoneum** *	No	91	reference	reference	reference	reference
	Yes	91	0.02	1.04	2.82	1.15 to 6.88
**Platelet group**	Very low (0-59K u/L)	30	0.69	-0.27	0.76	0.20 to 2.92
	Low (60K-117K u/L)	38	0.04	1.35	3.87	1.08 to 13.86
	Low normal (118K–249K u/L)	57	0.99	0.01	1.01	0.32 to 3.15
	Normal (250K-490K u/L)	44	reference	reference	reference	reference
	High (>490K u/L)	11	0.11	1.88	6.53	0.65 to 65.51
**Primary surgery**	No	33	reference	reference	reference	reference
	Yes	149	0.06	2.04	7.66	0.94 to 62.48

* significant P-value at P< 0.05.

## Discussion

This study documented the histopathologic distribution of canine splenic masses treated by splenectomy at a privately owned specialty practice. In the present study, HSA was identified in only 32.4% of the dogs. Malignant splenic lesions were identified in only 42.3% of the dogs, while the majority of splenic lesions, 57.7%, were benign. These results confirmed our hypotheses. The low frequency of malignant splenic lesions and HSA found in the current study, were similar to another study’s recent findings that only 27% of the dogs that underwent splenectomy surgery due to a splenic tumor, were malignant [15].

Guidelines regarding prognosis and likelihood of splenic malignancy are needed when making decisions with clients in private practice. The double two-thirds rule is an easily understandable and helpful tool that was established to better guide veterinarians and owners in making decisions when splenic lesions were diagnosed. This rule was established based on splenic histopathology from dogs with splenomegaly that underwent splenectomy surgery or necropsy at a veterinary teaching hospital [13]. The fifty-fifty rule is also sometimes used during clinical decision making, and this rule was based on a study that identified neoplastic lesions in 50% of the canine spleens submitted to a diagnostic laboratory, and approximately 50% of the neoplastic lesions were diagnosed as HSA [20]. Since these seminal studies, preoperative diagnostic tools have advanced, potentially leading to increased detection of splenic masses and nodules with and without concurrent splenomegaly. Breeds, longevity, and client expectations have also been changing [21,22]. Recent studies have therefore been reevaluating likelihood of splenic malignancy and HSA based on different subsets of criteria, and other factors are being considered in guidelines to assist in clinical decision-making [7,8,11,15,19,23,24]. Our study differed from other studies in that we included dogs that had both primary and incidental splenic masses with and without hemoperitoneum, and splenectomies were performed at a privately-owned specialty clinic.

This study identified four predictors (decreased PLT, decreased HCT, smaller diameter of largest splenic lesion, and presence of hemoperitoneum) of increased likelihood of an HSA diagnosis, and three of these (presence of hemoperitoneum, decreased HCT, and smaller diameter of largest splenic lesion) were also predictors for splenic malignancy including HSA. These risk factors were also similarly identified in multiple other studies [4,10,12,19,23,25,26].

Currently, preoperative diagnostics do not provide conclusive evidence regarding malignancy of splenic masses in dogs. Cytopathology is sometimes used to evaluate splenic masses prior to surgery; however, this diagnostic is not always useful nor recommended. Risks include the potential for iatrogenic mass rupture that may lead to abdominal hemorrhage and the seeding of tumor cells into the body wall. Additionally, the low exfoliating potential of HSA, hemangioma, and hematoma masses further complicates cytopathologic evaluation [13,27] and cytology results do not necessarily correlate with histopathologic findings for splenic masses; one study found that cytology correlated with histopathology only 61% of the time [28]. Another recent study found that cytology of canine splenic lesions had a high specificity of 95.5% but a low sensitivity of 64.3% and negative predictive value of 51.2%, indicating that cytology might incorrectly diagnose nearly half of malignant lesions as benign [29].

Hematologic abnormalities have been documented in dogs with HSA and splenic malignancies [8,12,25,30,31]. Specifically, thrombocytopenia and anemia have been found to be associated with a higher risk of perioperative death or shorter postoperative survival times in dogs undergoing splenectomies [19,32]. An early study [14] found that thrombocytopenia was the most common abnormality occurring in 75% of the dogs with HSA, and dogs with HSA had significantly lower PLT compared to dogs with other types of splenic masses; in addition, 25% of the dogs died due to hemostatic abnormalities [14]. Our study supported previous findings, as dogs with low PLT (60K-117K u/L) were 21.4 times more likely to have HSA when compared to dogs with normal PLT; furthermore, only four dogs with HSA in our study had normal or high platelet counts. Our study also found that dogs with low HCT (<33%) had 3.1 times the odds of an HSA diagnosis compared with dogs with a normal HCT. Previous studies [8,30] also have found that dogs diagnosed with HSA had significantly lower PCV and that PCV was higher in dogs with benign splenic masses [8]. Similarly, no dogs with HSA in our study had a high HCT. It is possible that some of our findings may be attributed to the presumption that malignant splenic masses might be more likely to rupture and cause intermittent or continuous hemorrhage causing anemia and thrombocytopenia.

In our study, there were 33 dogs that underwent a splenectomy when a splenic lesion was identified incidentally. Of the 33 incidental splenic lesions, 31 (93.9%) were benign. The findings from a recent study [7] that used records from both a university and specialty clinic aligned with the less common fifty-fifty rule [20]. That study [7], however, only evaluated dogs that presented with hemoperitoneum and/or clinical signs indicative of splenic disease; therefore, dogs that had splenic masses identified incidentally were likely not included in their study, and this might have led to the exclusion of additional benign splenic diagnoses. Furthermore, a recent study [8] evaluated only incidentally identified splenic masses in dogs without concurrent hemoperitoneum, and they found that 70.5% of these were benign. Considering the large majority of incidentally detected splenic masses diagnosed as benign lesions, prudence is warranted when performing splenectomies in these cases, as splenectomy itself can result in adverse events.

Other tests, algorithms, and risk factors have been explored to better assess risk of malignancy and HSA in splenic lesions [12,26,33,34]. Hemoperitoneum is often cited as one of the predictive factors, and incidence of malignancy for dogs with hemoperitoneum has been reported generally between 60%–80% [23,26,33,35], with the majority of the malignancies attributed to HSA. Our study also identified hemoperitoneum as one of the significant predictors of malignancy and HSA, as 60.4% of the 91 dogs with hemoperitoneum had a splenic malignancy including HSA. Another study evaluated specifically anemic dogs that received transfusions during treatment for hemoperitoneum or a splenic mass, and malignancy was identified in 76.1% of the dogs and 70.4% having HSA [12]. A recent meta-analysis confirmed those findings that over 70% of dogs with nontraumatic hemoperitoneum due to a splenic mass, had a malignant splenic lesion [24].

Splenic mass relative size and number of nodules have also been considered as predictors of malignancy and HSA. Our study did not find an association between number of nodules and risk of malignancy or HSA, and this lack of association was also found in another study that evaluated solitary versus multiple splenic lesions in dogs [33]. Our study found that size of the largest splenic lesion was associated with risk of HSA. As the largest splenic nodule increased in diameter, the dog’s risk of having an HSA diagnosis decreased; larger lesions were more likely to be benign. This corroborates other studies’ findings [10,17]. One study found that masses larger than 7 cm were more likely to be benign [17], and another found that benign splenic masses had significantly larger mass-to-spleen size ratios and increased splenic weight as a percentage of body weight as compared to malignant tumors [10]. These findings might be attributed to the potentially faster growth rate of smaller masses, which might initiate rupture more readily [17]. Malignant masses are also more likely to have disorganized architecture that predispose them to spontaneous rupture sooner compared to benign lesions [11]. Other studies, however, have not found an association between splenic mass size and histopathologic diagnosis or overall prognosis [13,20].

Historically, German shepherds (GSD), Retrievers, and Boxers were overrepresented in cases with hemangiosarcoma [1,4,13]. Recent studies have found associations with breed and weight or size of dog and likelihood of HSA or splenic malignancy [7,11,31]. One study found that smaller dogs were less likely to be diagnosed with HSA, but they did not find a significant difference in occurrence of splenic malignancy when comparing small versus large dogs; however their cutoff for small versus large dogs was 27.8 kg thereby including considerably large-sized dogs in their small category [31]. Other studies have found differences based on size and breed with large-breed dogs being more likely diagnosed with HSA and splenic malignancy or hemorrhage [7,11,36]. All 6 of the GSD in our study were diagnosed with HSA; however, the overall breed-size category was not a statistically significant predictor of HSA in our study. This could be due to small numbers of dogs in each breed such that most of the dogs (133/182, 73.1%) in our study were either mixed-breed or represented by a breed that had 5 or less dogs, thus those 133 dogs were assigned to the breed-size category based on size. This might also suggest that breeds differed in our study as compared to other studies due to geographic location or clinic type, or that breed demographics are changing and were represented by a large proportion of mixed breeds in our study. However, the breeds assigned in our study were based on owner-report without genetic analysis or breed documentation. This could have resulted in misrepresentation and inaccurate categorization of breeds. Furthermore, there were breed bans in one of the counties adjacent to the clinic in our study. The dogs that might have been phenotypically characterized as belonging to one of the banned breeds, might have instead been assigned a breed, as per owner report, that was not banned. These suppositions should not be undervalued and may be noteworthy considering other reports of breed predisposition to HSA or splenic malignancy.

The survival to discharge rate in this study was 91%. Of the 16 dogs that were not successfully discharged, 50% had a malignancy diagnosis, and 50% had benign disease. Dogs undergoing splenectomy have been shown to have hemorrhage-associated hemostatic dysfunction and post-operative thrombosis including portal system thrombosis and pulmonary thromboembolism [19]. Although some previous studies showed a low mortality rate in dogs with splenectomy due to splenic hematomas [18], a recent study found that dogs that underwent splenectomy due to benign splenic masses and hemoperitoneum, died prematurely at a high and clinically significant rate [17]. Considering the potential for adverse outcomes and complications leading to shortened survival times, further caution and an exhaustive exploration of risk factors for malignancy might be warranted when splenectomy decisions are made in dogs with splenic lesions or hemoperitoneum.

There were several limitations with our study. It was retrospective and relied on data collected from review of medical records. Some of the information was also therefore sometimes based on owner report such as breed and age of dog. Because of the retrospective nature and the sole inclusion of dogs that underwent splenectomy, dogs that were presented to the clinic for splenic masses or spleen-related clinical signs but did not undergo surgery, were not included. Such dogs could have been euthanized, died prior to or during surgery, or clients could have elected at-home medical management or palliative care. Inclusion of these groups could have resulted in different findings. Furthermore, if clients did not consent to sample submission and histopathology, particularly if their dog had died or been euthanized during surgery, those dogs could not be included in our study. The lack of inclusion of those dogs could have resulted in fewer malignant disease processes diagnosed. Future studies might consider prospectively evaluating splenic and other organ pathology of those dogs while removing the financial burden associated with histopathology submission. For those dogs that elected medical management and at-home care in lieu of surgery, future studies might consider following those dogs, outcome, survival, and histopathology post-mortem. Another limitation was that histopathology slides were read by multiple pathologists, and the slides were not available to be reviewed by the authors. Biopsies and histopathologic evaluation of the liver of the dogs were also not included; liver biopsies could have provided further information regarding malignancy [37]. Our study also did not include long-term follow-up of the dogs, thus overall outcome could not be assessed.

## Conclusions

Our study found that the majority of dogs that underwent splenectomy surgery had benign splenic lesions. Based on our findings that hemoperitoneum, anemia, thrombocytopenia, and smaller diameter of splenic lesions were risk factors for HSA especially, and the first three can be associated with hemorrhage, it is suggested that these associations be strongly considered when evaluating splenic masses and deciding to perform splenectomies. Furthermore, considering that the large majority (93.9%) of incidentally found splenic masses were benign, extra caution is warranted if splenectomy is being considered in a dog without clinical signs indicative of malignant splenic disease or clear risk factors for malignancy.

Overall prevalence of splenic malignancy including HSA may be overestimated in some canine populations if clinicians are evaluating non-ruptured splenic masses in dogs being seen in private practice. The information provided by our study may be useful for veterinarians when evaluating options, risk factors, and prognoses for HSA and the possibility of malignancy and when discussing the above with clients. Canine demographics, breed distributions, and longevity are continually changing, thus clinical patterns and diagnoses likely also continually change and differ from patterns established decades prior.
